# Paternal Contributions to Recurrent Pregnancy Loss: Mechanisms, Biomarkers, and Therapeutic Approaches

**DOI:** 10.3390/medicina60121920

**Published:** 2024-11-22

**Authors:** Aris Kaltsas, Athanasios Zikopoulos, Vladimir Kojovic, Fotios Dimitriadis, Nikolaos Sofikitis, Michael Chrisofos, Athanasios Zachariou

**Affiliations:** 1 Third Department of Urology, Attikon University Hospital, School of Medicine, National and Kapodistrian University of Athens, 12462 Athens, Greece; ares-kaltsas@hotmail.com (A.K.); mxchris@yahoo.com (M.C.); 2 Department of Obstetrics and Gynecology, Royal Cornwall Hospital, Truro TR1 3LJ, UK; athanasios.zikopoulos1@nhs.net; 3 Department of Urology, Faculty of Medicine, University of Belgrade, 11000 Belgrade, Serbia; kojovic.dr@gmail.com; 4 Department of Urology, Faculty of Medicine, School of Health Sciences, Aristotle University of Thessaloniki, 54124 Thessaloniki, Greece; helabio@yahoo.gr; 5 Laboratory of Spermatology, Department of Urology, Faculty of Medicine, School of Health Sciences, University of Ioannina, 45110 Ioannina, Greece; nsofikit@uoi.gr

**Keywords:** recurrent pregnancy loss, paternal factors, sperm DNA fragmentation, epigenetics, proteomics, male infertility, oxidative stress, lifestyle factors, environmental exposures

## Abstract

*Background and Objectives*: Recurrent pregnancy loss (RPL) affects numerous couples worldwide and has traditionally been attributed mainly to maternal factors. However, recent evidence highlights significant paternal influences on pregnancy viability and outcomes. This review aims to comprehensively examine male contributions to pregnancy loss, focusing on underlying mechanisms, novel biomarkers, and integrated strategies for improved reproductive success. *Materials and Methods*: A comprehensive narrative review was conducted by searching databases including PubMed and Embase for the literature published from January 2004 to October 2024. Studies focusing on paternal influences in RPL—encompassing oxidative stress, genetic and epigenetic mechanisms, health conditions, lifestyle factors, environmental exposures, and advancements in sperm proteomics—were included. Inclusion criteria were peer-reviewed articles in English that directly addressed paternal factors in RPL; studies not meeting these criteria were excluded. *Results*: The review identified that paternal factors such as advanced age, metabolic and cardiovascular health issues, chronic diseases, lifestyle habits (e.g., smoking, alcohol consumption, poor diet), and environmental exposures significantly affect sperm integrity through mechanisms like oxidative stress, DNA fragmentation, and epigenetic alterations. Advanced paternal age and poor health conditions are associated with increased risks of miscarriage and adverse pregnancy outcomes. Novel sperm proteomic biomarkers have been identified, offering potential for enhanced diagnostics and personalized interventions. Integrated approaches involving multidisciplinary assessments, preventive strategies, and genetic counseling are essential for effectively addressing RPL. *Conclusions*: Integrating paternal factors into clinical evaluations is crucial for effectively addressing recurrent pregnancy loss. Recognizing and modifying paternal risk factors through lifestyle changes, medical interventions, and environmental management can improve pregnancy outcomes. The findings underscore the need for incorporating paternal assessments into standard care and highlight the importance of future research focusing on standardizing diagnostic protocols, expanding studies on paternal contributions, and integrating proteomic biomarkers into clinical practice to facilitate personalized treatment strategies.

## 1. Introduction

Pregnancy loss is a profoundly distressing experience that affects a significant proportion of couples attempting to conceive, with miscarriage occurring in approximately 15–25% of clinically recognized pregnancies. Among these, recurrent pregnancy loss (RPL)—defined as the loss of two or more consecutive pregnancies—impacts about 5% of women, while around 1% experience three or more successive losses [1,2]. Traditionally, diagnostic evaluations for pregnancy loss have predominantly focused on maternal factors such as uterine abnormalities, hormonal imbalances, infections, immunological disorders, and chromosomal anomalies. Advanced maternal age, in particular, has been consistently identified as a significant risk factor for pregnancy loss [3].

In contrast, paternal factors have historically received limited attention in clinical assessments of pregnancy loss [4]. Despite increasing evidence highlighting the importance of male contributions, current clinical guidelines often recommend only limited testing for paternal factors, such as karyotype analysis and sperm DNA fragmentation assessments, to evaluate male etiology [5,6]. Recent research indicates that paternal influences may be critical, affecting both pregnancy viability and overall reproductive success [7]. Factors including advanced paternal age, lifestyle choices, health status, genetic composition, and sperm quality have been significantly associated with the risk of miscarriage and fetal abnormalities [8,9].

Given the expanding body of literature illuminating the paternal impact on pregnancy outcomes, there is a clear imperative to integrate male factors into the evaluation of pregnancy loss. This review aims to comprehensively examine male contributions to pregnancy loss, focusing on paternal age, genetic factors, overall health, lifestyle influences, and sperm quality. By addressing these areas, we seek to provide new insights into potentially modifiable paternal risk factors, broaden the scope of pregnancy loss evaluation, and foster improved pregnancy outcomes.

## 2. Materials and Methods

### 2.1. Literature Search Strategy

A comprehensive literature search was performed to gather relevant studies on paternal contributions to RPL. The databases searched included PubMed, Embase, Scopus, and Web of Science, covering publications from January 2004 to October 2024. This time frame was chosen to capture the most recent and significant developments in the field. The search utilized a combination of keywords and Medical Subject Headings (MeSH) terms related to RPL and paternal factors. The search terms included:

- “Recurrent pregnancy loss” OR “recurrent miscarriage” OR “spontaneous abortion”
- “Paternal factors” OR “male infertility” OR “sperm quality” OR “sperm DNA fragmentation”
- “Oxidative stress” OR “genetic mechanisms” OR “epigenetic alterations”
- “Advanced paternal age” OR “paternal health” OR “lifestyle factors” OR “environmental exposures”
- “Proteomics” OR “biomarkers” OR “sperm proteins”

Boolean operators (AND, OR) were employed to refine the search and ensure a comprehensive retrieval of the relevant literature.

### 2.2. Inclusion and Exclusion Criteria

Inclusion Criteria:

- Peer-reviewed articles published in English.
- Original research studies, systematic reviews, meta-analyses, and clinical guidelines focusing on paternal factors influencing RPL.
- Studies addressing mechanisms that may impact sperm quality, including oxidative stress; genetic and epigenetic alterations; health conditions (e.g., chronic diseases, metabolic syndrome); lifestyle factors; environmental exposures (e.g., pollutants, occupational hazards); medications; and advancements in sperm proteomics.
- Human studies involving adult male participants contributing to pregnancy loss.

Exclusion Criteria:

- Non-English publications.
- Studies not directly related to paternal factors in RPL, such as those exclusively examining maternal factors.
- Case reports, editorials, commentaries, and conference abstracts without full-text availability.
- Animal studies, unless they provided significant insights translatable to human physiology.
- Studies published prior to 2004, unless considered seminal works crucial for background information.

### 2.3. Study Selection Process

The titles and abstracts of the remaining studies were screened independently by two reviewers (A.K. and A.Z.) to ensure adherence to inclusion criteria, with studies failing to meet the inclusion criteria excluded at this stage. Full-text articles of potentially relevant studies were then retrieved and assessed for eligibility. Any discrepancies in study selection were resolved through consensus discussions between the reviewers. In cases where a consensus could not be achieved, a third reviewer (V.K.) was consulted to ensure methodological rigor and reduce potential bias in study selection.

### 2.4. Data Extraction and Synthesis

Data were extracted from the selected studies using a standardized data extraction form. The extracted information included:

- Study characteristics: author(s), year of publication, study design, sample size, and population demographics.
- Key findings: results related to paternal factors influencing RPL.
- Mechanisms explored: details on oxidative stress, genetic mutations, epigenetic modifications, and other relevant mechanisms.
- Identified biomarkers: information on novel sperm proteomic biomarkers and their clinical implications.
- Recommendations: suggestions for clinical practice and future research directions provided by the studies.

Given the heterogeneity of the included studies in terms of design and outcomes, a narrative synthesis was conducted. This approach allowed for the integration of findings across different study types and the identification of common themes and patterns related to paternal contributions to RPL.

## 3. Mechanisms of Paternal Influence on Pregnancy Loss

### 3.1. Oxidative Stress and Sperm Integrity

Oxidative stress, marked by an imbalance of reactive oxygen species (ROS), significantly affects male fertility by compromising sperm integrity. Environmental pollutants, smoking, lifestyle factors, and conditions like varicocele contribute to excessive ROS generation. Elevated ROS levels damage sperm DNA and proteins, leading to chromatin instability and DNA fragmentation, which in turn compromise embryo viability and increase the risk of pregnancy loss [10,11]. Additionally, oxidative stress induces lipid peroxidation and protein carbonylation in spermatozoa, potentially disrupting early embryonic development by altering paternal genetic and epigenetic information [11].

### 3.2. Genetic Mechanisms

Genetic factors play a crucial role in paternal contributions to RPL, primarily through sperm DNA fragmentation and chromosomal abnormalities.

#### 3.2.1. Sperm DNA Fragmentation

Sperm DNA fragmentation is a key indicator of compromised genetic integrity. As sperm cells mature and transit through the male reproductive tract, levels of DNA fragmentation can increase due to oxidative stress and environmental damage, particularly affecting DNA regions less protected by protamines [12,13,14]. Elevated sperm DNA fragmentation has been consistently linked to adverse pregnancy outcomes, including RPL and miscarriage [15,16].

The impact on embryo development involves several mechanisms:

- Impaired Fertilization and Zygote Viability: fragmented sperm DNA disrupts chromatin stability, hindering the proper merging of paternal and maternal genomes during fertilization. This can lead to defective early cell divisions and genomic activation, resulting in embryo arrest or early pregnancy loss [5,12].
- Oxidative Stress and Epigenetic Alterations: oxidative stress-induced DNA fragmentation can cause abnormal DNA methylation patterns. These epigenetic changes may be transmitted to the embryo, disrupting gene regulation essential for normal development and increasing the risk of RPL [17,18].
- Overwhelmed Embryonic Repair Mechanisms: while embryos have DNA repair systems to address paternal DNA damage, excessive fragmentation can exceed their capacity, leading to cell cycle defects and apoptosis. This reduces the likelihood of the embryo progressing to the blastocyst stage or establishing a successful pregnancy [19,20].

Meta-analyses support the association between elevated sperm DNA damage and increased miscarriage risk. Robinson et al. (2012) reported a significant correlation, with a relative risk of 2.16 for couples with higher DNA damage McQueen et al. (2019) found that male partners of women with RPL had significantly higher DNA fragmentation compared to partners of fertile women [21]. A 2021 meta-analysis further confirmed this association, highlighting the importance of sperm DNA integrity in pregnancy outcomes [20]. Interventions aimed at reducing sperm DNA fragmentation, such as antioxidant therapy, lifestyle modifications, and surgical treatments like varicocelectomy, have shown promise in improving sperm quality and reducing the incidence of RPL [22,23].

#### 3.2.2. Chromosomal Abnormalities

Chromosomal abnormalities, particularly translocations, significantly increase the risk of miscarriage [24]. Robertsonian translocations, one of the most common types, involve the fusion of two acrocentric chromosomes, leading to genetically unbalanced gametes [25,26]. Male carriers may produce sperm with extra or missing genetic material, resulting in embryos with chromosomal imbalances that disrupt normal development and frequently lead to miscarriage. Approximately 2.9% of couples experiencing two or more pregnancy losses have chromosomal abnormalities, including such translocations [27].

To address chromosomal abnormalities, guidelines recommend that male partners in couples affected by RPL undergo karyotyping to detect any chromosomal rearrangements [28]. If identified, assisted reproductive technologies like intracytoplasmic sperm injection (ICSI) combined with preimplantation genetic diagnosis (PGD) can select embryos without chromosomal abnormalities before implantation, thereby enhancing the likelihood of a successful pregnancy. However, these interventions do not entirely eliminate the risk of pregnancy loss due to chromosomal issues [29].

#### 3.2.3. Inherited Genetic Mutations

Microdeletions in the Y chromosome, specifically in regions AZFa, AZFb, and AZFc, are associated with male infertility and an increased risk of RPL [30]. These deletions, collectively referred to as azoospermia factors (AZF), appear in approximately 3–5% of men with oligozoospermia and 6–16% of those with azoospermia, contributing to deficient sperm quality and compromised embryo development [31]. Dewan et al. (2006) conducted one of the initial studies suggesting a strong link between Y-chromosome microdeletions and RPL, finding that 82% of the male partners in RPL cases had AZFc microdeletions [32]. Subsequent studies, such as those by Karaer et al. (2015) and Agarwal et al. (2015), supported this connection, reporting similar prevalence rates of microdeletions among men in couples with RPL [33,34].

However, research on this topic presents mixed results. Some studies, including those by Wettasinghe et al. (2010), Ghorbian et al. (2012), and Venkatesh et al. (2011), did not find significant associations between Y-chromosome microdeletions and RPL, highlighting the complexity of pregnancy loss and suggesting that both genetic and environmental factors may jointly influence these outcomes [35,36,37]. Consequently, while Y-chromosome microdeletion testing may provide valuable insights, further research is needed to clarify the role of these genetic abnormalities in RPL and to understand their interactions with other factors influencing reproductive success.

### 3.3. Epigenetic Mechanisms

Epigenetic alterations in sperm, including DNA methylation, histone modifications, and non-coding RNAs, play a significant role in embryonic development and pregnancy outcomes.

#### 3.3.1. DNA Methylation

Paternal age and health can significantly alter DNA methylation patterns in sperm, particularly at CpG-rich regions, impacting early embryonic development. Abnormal DNA methylation can disrupt gene expression, compromising embryo viability and increasing pregnancy complications. Environmental and lifestyle factors, such as smoking and diet, are known to influence methylation patterns, contributing to adverse reproductive outcomes. Recent studies further indicate that differentially methylated regions in sperm DNA correlate with fertility issues and unexplained RPL, suggesting a role for variable DNA methylation patterns in early embryogenesis defects [17,18].

#### 3.3.2. Histone Modification

Histone proteins in sperm play a critical role in chromatin structure, enabling or restricting access to genetic material and thus influencing gene expression. During sperm maturation, most histones are replaced by protamines to compact DNA, but some retain modifications that affect embryo development post-fertilization. Studies indicate that environmental factors, such as oxidative stress, can cause histone modifications like methylation or acetylation errors, impacting gene regulation necessary for healthy embryo development. These epigenetic changes can be transmitted across generations, affecting offspring health [38].

#### 3.3.3. Non-Coding RNAs

Non-coding RNAs (ncRNAs) in sperm, including microRNAs and tRNA-derived small RNAs, have essential roles in gene expression and early embryonic development. Extracellular vesicles from seminal plasma carry these RNAs, which influence maternal immune responses and fetal development. Research suggests that environmental influences, such as paternal diet and stress, can alter the ncRNA content in sperm, potentially affecting metabolic and neurodevelopmental outcomes in offspring [39,40].

Collectively, these mechanisms underscore the importance of paternal health in pregnancy outcomes and the potential transgenerational effects of environmental exposures on offspring development, highlighting the need for comprehensive paternal assessments in reproductive planning.

## 4. Paternal Factors Influencing Genetic and Epigenetic Mechanisms

Recent research highlights the significant impact of paternal health on pregnancy outcomes, particularly regarding the risk of pregnancy loss. While maternal health has traditionally been the focal point, emerging studies reveal that paternal factors—such as metabolic syndrome, advanced age, and lifestyle choices—play a crucial role in fetal development and viability [41].

Studies confirm that paternal metabolic syndrome—which includes obesity, diabetes, and hypertension—is independently associated with higher rates of pregnancy loss. Kasman et al. (2021), for instance, analyzed nearly one million pregnancies in the United States and demonstrated a stepwise relationship between the number of metabolic syndrome components in fathers and the risk of pregnancy loss, even after adjusting for maternal health factors [42].

Beyond direct metabolic effects, paternal health influences epigenetic changes in sperm that can be transmitted to offspring. Metabolic disorders in fathers alter the sperm epigenome, leading to disruptions in placental function and increasing the potential for metabolic diseases in the offspring later in life [43]. Pépin et al. (2022) further illustrated that paternal obesity affects the placental transcriptome, influencing fetal growth and development [44].

Advanced paternal age has also emerged as a significant risk factor for adverse pregnancy outcomes. As men age, accumulated DNA mutations and epigenetic alterations in sperm contribute to an elevated risk of fetal death and developmental issues in offspring, underscoring the importance of considering paternal age in reproductive counseling [45].

Lifestyle factors such as smoking, poor diet, and low physical activity have been linked to detrimental changes in sperm that increase the offspring’s risk for various health conditions. Interventions aimed at improving paternal health prior to conception—such as weight loss, smoking cessation, and increased physical activity—can significantly reduce these risks, highlighting the necessity of comprehensive health counseling for prospective fathers [46,47].

These findings represent a pivotal shift in understanding paternal health’s role in pregnancy outcomes. It is essential to consider both parents’ health when planning for pregnancy to enhance the likelihood of a healthy offspring.

### 4.1. Advanced Paternal Age

The established link between maternal age and adverse pregnancy outcomes, such as miscarriage, has been extensively documented, with risks increasing notably after the age of 35 [48]. A comprehensive Norwegian study encompassing 421,201 pregnancies found that miscarriage rates were lowest among women aged 25 to 29 years (10%) but rose markedly with advancing age, reaching 53% in women aged 45 or older. Moreover, a history of prior miscarriage heightened the risk of subsequent loss by 50% [49].

In recent years, advanced paternal age has also been recognized as a significant factor influencing pregnancy outcomes. As men contribute half of the genetic material to the embryo, the age of the father may critically affect cases of RPL. A systematic review and meta-analysis conducted by du Fossé et al., analyzing ten population-based cohort and case-control studies, identified a clear association between paternal age over 40 and an increased risk of spontaneous miscarriage, independent of maternal age [50]. Specifically, fathers aged 40 to 44 exhibited a 23% higher likelihood of miscarriage before 20 weeks of gestation compared to younger fathers. This risk escalated with advancing age: men over 45 years faced a 43% increase in miscarriage risk before 20 weeks and a 74% increase before 13 weeks [50].

Several biological mechanisms have been proposed to explain the link between advanced paternal age and RPL:

- Increased De Novo Mutations: as men age, the number of cell divisions during spermatogenesis accumulates, leading to a higher rate of spontaneous genetic mutations, or de novo mutations, in sperm DNA. It is estimated that the frequency of these new mutations doubles approximately every 16 years of paternal age [7]. Such mutations can disrupt essential genes in the embryo, increasing the likelihood of genetic abnormalities that may result in miscarriage [51,52].
- Telomere Shortening: telomeres are protective nucleotide sequences at the ends of chromosomes that shorten with each cell division. While sperm telomere length may initially increase up to around age 40 as a compensatory mechanism, research indicates that telomere length begins to decrease in older age [34]. Shortened telomeres in sperm are associated with chromosomal instability, which can compromise embryo viability and elevate the risk of miscarriage [53,54].
- Decline in Sperm Quality and DNA Integrity: advanced paternal age is associated with a decline in overall sperm quality, including reduced motility and abnormal morphology. Importantly, the sperm fragmentation index (DFI) and the extent of DNA damage can increase significantly with age, potentially doubling between the ages of 20 and 60 [55]. Elevated DNA fragmentation undermines the genetic stability of the embryo, contributing to embryonic loss due to genetic abnormalities [56].

These mechanisms demonstrate how genetic and epigenetic alterations in the sperm of older men can adversely affect embryo development and pregnancy outcomes. In addition to miscarriage, advanced paternal age has been associated with other complications, such as preterm birth, low birth weight, congenital anomalies, and an increased incidence of developmental disorders in offspring, including autism spectrum disorders and schizophrenia, particularly when paternal age exceeds 50 years [9].

Recognizing advanced paternal age as a potential risk factor is crucial when evaluating patients with RPL or unexplained infertility. Clinicians should provide appropriate counseling to couples, especially those with idiopathic RPL, to support informed decision-making in family planning.

### 4.2. Health Conditions

Paternal health conditions, particularly metabolic and cardiovascular diseases like obesity and diabetes, have been increasingly recognized for their adverse effects on pregnancy outcomes. These conditions contribute to RPL through mechanisms such as epigenetic modifications in sperm and increased oxidative stress leading to genetic instability [57,58].

#### 4.2.1. Obesity-Induced Epigenetic Changes

Obesity in men has been associated with alterations in sperm DNA methylation patterns and histone modifications—key epigenetic mechanisms that regulate gene expression without changing the underlying DNA sequence. For instance, obesity can lead to hypermethylation or hypomethylation of genes involved in essential developmental pathways. Hyper-methylation may silence genes critical for normal embryonic growth, while hypo-methylation might activate transposable elements or oncogenes, disrupting genomic stability [59,60].

Studies have demonstrated that obese fathers exhibit differential methylation in imprinted genes—genes where only one allele is expressed depending on the parent of origin—which can affect placental function and fetal growth [61]. Additionally, obesity can disrupt histone acetylation patterns, altering chromatin structure and affecting sperm chromatin packaging, potentially leading to aberrant gene expression in the embryo.

#### 4.2.2. Diabetes-Related Epigenetic Impact

Paternal diabetes influences sperm epigenetics through chronic hyperglycemia and increased production of ROS. Elevated glucose levels can affect the expression of DNA methyltransferases (DNMTs), enzymes responsible for adding methyl groups to DNA, resulting in abnormal methylation patterns [62]. Moreover, the oxidative stress associated with diabetes can cause oxidative DNA damage, leading to mutations and epigenetic alterations in sperm DNA [63]. For example, oxidative lesions like 8-oxo-2′-deoxyguanosine can mispair during DNA replication, potentially introducing mutations that may be transmitted to the offspring [64]. These epigenetic disruptions interfere with normal embryonic development, increasing the risk of miscarriage.

#### 4.2.3. Cardiovascular Conditions and RPL Risk

Cardiovascular diseases, such as hypertension, further heighten the risk of RPL by inducing systemic inflammation and endothelial dysfunction, which negatively affect sperm quality. Inflammatory cytokines can alter the testicular microenvironment, impairing spermatogenesis and leading to epigenetic changes in sperm DNA [65,66]. These alterations may impact genes involved in vascular development and placental formation, contributing to impaired placental function and fetal loss. Additionally, the combination of advanced paternal age and cardiovascular conditions has been associated with increased sperm DNA fragmentation and chromosomal abnormalities, further elevating the risk of RPL [67].

#### 4.2.4. Chronic Diseases: Influence on Oxidative Stress and Genetic Stability

Chronic paternal conditions, particularly those contributing to oxidative stress, play a crucial role in genetic stability and the risk of RPL. Conditions such as diabetes and obesity are closely associated with heightened levels of oxidative stress, leading to DNA damage in sperm cells. This oxidative stress disrupts sperm integrity by impairing DNA repair mechanisms, which may increase the risk of chromosomal abnormalities in embryos and contribute to early pregnancy loss. Elevated oxidative stress is often observed in men with metabolic disorders, where ROS damage not only sperm DNA but also alter key epigenetic regulators that affect embryonic viability [68].

Genetic instability due to oxidative stress is particularly significant for RPL. Studies have shown that sperm from men with chronic diseases exhibits higher rates of DNA fragmentation and epigenetic abnormalities. Such genetic alterations are linked to an increased likelihood of miscarriage, likely due to impaired placental function and disrupted fetal development. Chronic disease management and reduction of oxidative stress through lifestyle interventions have been proposed to improve sperm quality, which may reduce the incidence of RPL and support healthier pregnancy outcomes [69].

### 4.3. Lifestyle Factors

Specific paternal lifestyle factors significantly influence pregnancy outcomes, including RPL. Smoking, alcohol consumption, physical activity, and diet each contribute uniquely to sperm quality and genetic stability, often through mechanisms involving oxidative stress, DNA damage, hormonal disruption, or epigenetic alterations.

#### 4.3.1. Smoking and DNA Damage

Paternal smoking has been strongly linked to impaired sperm quality, contributing to increased DNA fragmentation and epigenetic alterations. Cigarette smoke contains numerous harmful compounds, including ROS and toxins, which generate oxidative stress and cause structural DNA damage in sperm cells. This fragmentation compromises the genetic integrity required for healthy embryonic development. Smoking also induces epigenetic changes, such as alterations in DNA methylation patterns and non-coding RNA expression in sperm, potentially affecting gene regulation in the embryo. These modifications are associated with disrupted placental function and increased pregnancy complications, making smoking a key modifiable risk factor for RPL [70].

#### 4.3.2. Alcohol Consumption: Impact on Sperm DNA and Hormonal Balance

Alcohol consumption impacts sperm quality through mechanisms that induce DNA damage and hormonal imbalance. Chronic alcohol intake increases oxidative stress, leading to sperm DNA fragmentation and reduced sperm motility. Additionally, alcohol can alter hormone levels, particularly testosterone, which is crucial for normal sperm production and function. Hormonal imbalances disrupt spermatogenesis, resulting in lower sperm counts and increased morphological abnormalities, all of which contribute to higher RPL risk. Limiting alcohol consumption prior to conception is recommended to minimize these risks [19].

#### 4.3.3. Physical Activity

Moderate physical activity is generally beneficial for reproductive health, enhancing cardiovascular function, reducing stress, and improving metabolic health—all supporting healthy sperm production. However, excessive or intense physical activity can have adverse effects, particularly through increased oxidative stress and hormonal disruptions. High-intensity exercise may elevate cortisol levels, leading to reduced testosterone and altered sperm parameters, potentially increasing the risk of RPL. Optimal levels of physical activity—such as moderate aerobic exercises combined with strength training—are associated with better sperm quality, whereas overtraining can be detrimental to reproductive outcomes [71].

#### 4.3.4. Diet and Nutrition

Diet plays a crucial role in determining sperm quality. A nutrient-rich diet high in antioxidants is beneficial for reducing oxidative stress and enhancing genetic stability in sperm. Antioxidants such as vitamins C and E, selenium, and zinc help neutralize ROS, reducing DNA fragmentation in sperm. Diets rich in these nutrients are associated with improved sperm motility, morphology, and lower rates of DNA damage, which can lower RPL risk. In contrast, poor dietary habits—particularly high intake of processed foods and trans fats—contribute to metabolic imbalance and oxidative stress, impairing sperm quality. Preconception nutritional counseling to improve dietary intake of antioxidants can be a key intervention to support healthy pregnancy outcomes [72,73,74].

Addressing paternal lifestyle factors is essential in reducing the incidence of RPL and improving overall reproductive health. Modifiable behaviors such as smoking cessation, limiting alcohol intake, maintaining balanced physical activity, and prioritizing antioxidant-rich diets offer protective benefits that support sperm integrity and mitigate RPL risk.

### 4.4. Environmental Exposures

Environmental exposures are increasingly recognized as critical factors influencing male reproductive health and the risk of RPL. Exposure to toxins, pollutants, and certain occupational hazards can induce harmful changes in sperm, affecting DNA integrity and epigenetic stability, ultimately impacting pregnancy outcomes.

#### 4.4.1. Toxins and Pollutants

Exposure to environmental toxins and pollutants—such as heavy metals, pesticides, air pollution, and endocrine-disrupting chemicals (EDCs)—leads to significant damage in sperm DNA and alters epigenetic patterns. These toxins induce oxidative stress, generating ROS that damage sperm DNA, leading to fragmentation and chromosomal abnormalities. Epigenetic alterations, such as DNA methylation and histone modifications, are observed in response to toxin exposure, particularly affecting genes associated with fetal development and placental function. Pollutants like bisphenol A (BPA), phthalates, and lead are known to disrupt hormone levels and interfere with epigenetic regulatory processes, further increasing the risk of embryonic development issues and pregnancy loss [43].

Research highlights the potential of environmental pollutants to introduce epigenetic “errors” in sperm that may be inherited across generations. For example, exposure to air pollutants such as fine particulate matter (PM2.5) has been shown to affect sperm methylation profiles, altering gene expression related to cellular growth and fetal viability. Such epigenetic disruptions are implicated in a range of adverse pregnancy outcomes, including miscarriage and preterm birth, emphasizing the need to minimize exposure to environmental pollutants in men of reproductive age [47].

#### 4.4.2. Occupational Hazards

Certain occupations pose specific reproductive risks for men, particularly those involving regular exposure to chemicals, radiation, or physical hazards. Jobs in industries such as agriculture, manufacturing, and healthcare may expose workers to pesticides, heavy metals, solvents, and ionizing radiation—all linked to poorer sperm quality and increased risk of genetic abnormalities. For instance, agricultural workers exposed to pesticides have shown higher rates of DNA fragmentation in sperm and altered sperm motility, factors that contribute to RPL risk. Exposure to radiation in healthcare professions has been associated with DNA strand breaks in sperm, leading to compromised genetic stability [70].

Heat exposure, common in professions such as welding or foundry work, impairs spermatogenesis by raising scrotal temperature, potentially reducing sperm count and quality. Studies suggest that men in high-exposure occupations experience a greater incidence of chromosomal anomalies in sperm, increasing the likelihood of early pregnancy loss. Awareness of occupational reproductive risks and implementing protective measures—such as using personal protective equipment (PPE) and ensuring proper ventilation—are essential for mitigating the impact of workplace hazards on reproductive health [71].

Environmental exposures to toxins, pollutants, and occupational hazards have profound impacts on paternal reproductive health, elevating the risk of RPL through DNA damage and epigenetic disruption in sperm. Minimizing these exposures through preventive strategies is critical in reducing RPL risks and promoting healthier pregnancy outcomes.

## 5. Sperm Proteomics and Novel Biomarkers

The field of proteomics has revealed significant insights into the roles of sperm proteins in fertility and embryonic development. Proteomics, the large-scale study of proteins, enables detailed analysis of protein alterations within sperm and their association with reproductive outcomes, including RPL. This section delves into the role of specific sperm proteins in fertility, emerging biomarkers, challenges in proteomics, and the clinical implications of these discoveries.

### 5.1. Role of Sperm Proteins in Fertility and Embryo Development

Recent studies underscore the vital role of specific sperm proteins in fertilization and early embryo development. Sperm proteins are essential for sperm motility, acrosome reaction, and successful fusion with the oocyte—all key processes in fertilization. These proteins also contribute to the subsequent stages of embryonic development, where they influence cell division and genomic stability. Variations in specific proteins, whether elevated or deficient, have been closely linked to RPL [75]. For example, increased levels of proteins such as ATP citrate lyase, DEAD-box helicase 1, fatty acid synthase, and Histone 1.2 are associated with RPL, suggesting that their overexpression may disrupt normal fertilization or early development processes. Conversely, reduced levels of proteins like Hexokinase 1 (HK1) and RuvB-like helicase have been observed in men experiencing RPL, indicating that these deficiencies may impair essential cellular functions during fertilization [47].

### 5.2. Identification of Novel Biomarkers for Reproductive Health

Proteomic analysis has identified a range of sperm proteins that may serve as biomarkers for assessing male fertility and RPL risk. These biomarkers, derived from protein expression profiles, provide a means of predicting reproductive outcomes based on the specific proteomic signature present in sperm. Elevated levels of ATP citrate lyase, for example, could serve as a predictive marker for RPL due to its association with disrupted energy metabolism in sperm. In contrast, low levels of HK1 may indicate compromised sperm function, offering a potential diagnostic tool for identifying men at higher risk for RPL. These biomarkers represent a promising avenue for improving diagnosis and treatment of male infertility, paving the way for personalized reproductive health assessments [76,77]. The Table 1 below summarizes key biomarkers identified in sperm proteomics, highlighting their functions and potential clinical implications for reproductive health.

### 5.3. Challenges and Future Directions in Proteomics

While proteomic research has advanced our understanding of sperm biology, several challenges remain in translating these findings into clinical practice. The variability in proteomic profiles among individuals, as well as the complex interactions within sperm regulatory networks, presents a significant hurdle in identifying universal biomarkers. Additionally, the need for standardization in protein quantification and analysis techniques is critical for reliable biomarker discovery. Future directions in sperm proteomics involve refining these methodologies, particularly by integrating proteomic data with other ‘omics’ approaches, such as genomics and epigenomics, to develop comprehensive reproductive health profiles. Advancements in high-throughput proteomics technology are also expected to enhance the sensitivity and specificity of biomarker identification, allowing for more accurate diagnoses of male infertility and RPL risks [78].

### 5.4. Clinical Implications

The identification of sperm proteomic biomarkers holds considerable clinical implications for male reproductive health. By targeting specific protein abnormalities, clinicians may gain new insights into previously unexplained cases of RPL, allowing for more tailored treatment approaches. For instance, therapies aimed at regulating the expression of proteins like ATP citrate lyase or DEAD-box helicase 1 could potentially reduce RPL risk. Moreover, the application of sperm proteomic profiles in fertility clinics could help identify men who may benefit from specific interventions, such as antioxidant supplementation or lifestyle changes, to improve sperm quality before conception. In the future, integrating proteomic biomarkers into routine fertility assessments may revolutionize male infertility diagnosis and management, promoting healthier pregnancy outcomes [79].

## 6. Integrated Approaches to Addressing Pregnancy Loss

Addressing RPL requires a comprehensive, multidisciplinary approach that considers the complex interplay of genetic, environmental, and lifestyle factors influencing both maternal and paternal health. Integrated strategies involving collaborative assessment, preventative and therapeutic interventions, and personalized genetic counseling are essential for enhancing pregnancy outcomes and supporting couples through fertility challenges [80].

### 6.1. Collaborative Assessment

A collaborative assessment model integrates the expertise of reproductive endocrinologists, geneticists, urologists, and mental health professionals to provide a thorough evaluation of both parents’ health. Given that paternal factors, including metabolic health, lifestyle, and environmental exposures, play a critical role in RPL, assessments should be inclusive of male health parameters. This collaboration enables a holistic view of RPL risk factors, including sperm DNA integrity, proteomic biomarkers, and genetic stability, as well as maternal reproductive health. Collaborative models also emphasize the role of advanced diagnostics, such as proteomics, genomics, and imaging techniques, to create a tailored profile for each couple, allowing for a more precise understanding of potential underlying causes [81].

### 6.2. Prevention and Intervention Strategies

Prevention and intervention strategies for RPL involve both lifestyle modifications and medical treatments aimed at addressing identified risk factors. For paternal health, recommended interventions may include smoking cessation, reduction in alcohol consumption, balanced nutrition, and moderate physical activity to improve sperm quality. Medical interventions, such as antioxidant therapy, may be suggested for men with high levels of oxidative stress to reduce DNA fragmentation in sperm. Recent studies indicate that dietary supplements, particularly those rich in antioxidants (e.g., vitamins C and E, selenium), can improve sperm DNA integrity, thereby reducing RPL risk [82,83,84].

Medical interventions can also address specific metabolic or cardiovascular health concerns, such as managing hypertension or diabetes in prospective fathers, to lower RPL risk [85]. For couples with identified genetic or epigenetic markers associated with RPL, more targeted interventions—such as in vitro fertilization (IVF) with preimplantation genetic testing—can help minimize the risk of pregnancy loss by selecting embryos without chromosomal abnormalities [86].

### 6.3. Genetic Counseling and Testing

Genetic counseling plays a crucial role in supporting couples experiencing RPL, offering insights into potential hereditary factors and guiding decisions about genetic testing. For both partners, genetic counseling includes an assessment of family history and risk factors for chromosomal abnormalities or inherited conditions that may contribute to RPL. Through counseling, couples can make informed decisions about available genetic testing options, such as karyotyping, single nucleotide polymorphism (SNP) testing, or next-generation sequencing, to identify genetic mutations or structural abnormalities [87].

For men, genetic counseling may involve testing for common genetic issues linked to male infertility, including Y-chromosome microdeletions or mutations in genes related to sperm production and function. Couples may also explore preconception genetic screening to identify and manage inheritable conditions early. As proteomics advances, incorporating proteomic biomarkers alongside genetic tests could provide even more comprehensive information about genetic and epigenetic risk factors, further enabling personalized intervention strategies [88].

In summary, an integrated approach combining collaborative assessment, preventative and therapeutic strategies, and genetic counseling provides a robust framework for addressing RPL. By considering both maternal and paternal factors, this comprehensive model offers a pathway to more personalized, effective treatment plans aimed at improving pregnancy outcomes and supporting the emotional and reproductive health of couples facing RPL.

## 7. Limitations and Future Research

**Figure 1:**
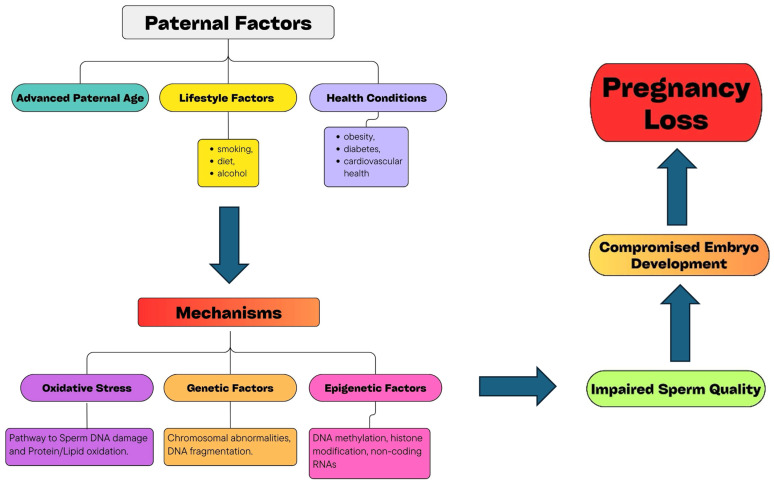
Paternal influences on reproductive health.

While significant progress has been made in understanding the factors contributing to RPL, there are still limitations in the current body of research and clinical approaches. Addressing these limitations and pursuing future research directions is crucial for developing more effective diagnostic tools, interventions, and support mechanisms for couples experiencing RPL. The following Figure 1 illustrates the various paternal factors and biological mechanisms that influence reproductive health and pregnancy outcomes, highlighting areas where further research and clinical focus are needed.

One primary strength of this review is its comprehensive scope. By examining paternal contributions to RPL through a wide lens, including genetic, epigenetic, lifestyle, and environmental influences, it provides clinicians and researchers with a well-rounded understanding of male factors that can affect reproductive outcomes. This holistic approach is intended to foster a more balanced consideration of both maternal and paternal factors in RPL, which could ultimately lead to improved diagnostic accuracy and intervention strategies. Additionally, the integration of emerging fields, such as proteomics and microbiome research, brings forward innovative diagnostic perspectives that highlight the potential of novel biomarkers. This inclusion emphasizes that new insights from these fields could complement traditional maternal-focused assessments, marking a shift toward a more integrated approach in reproductive health.

A further strength lies in the review’s focus on modifiable risk factors. By discussing lifestyle and environmental factors that influence paternal health, this work supports the development of targeted interventions that can be implemented in clinical settings. Preventable or treatable factors, such as smoking, diet, and exposure to environmental toxins, are emphasized as actionable areas where lifestyle modifications could play a significant role in reducing RPL risk. This focus offers both clinicians and couples practical strategies for improving reproductive outcomes, underscoring the importance of paternal health in the context of conception and pregnancy.

### 7.1. Limitations in Current Research and Clinical Approaches

One of the primary limitations in RPL research is the underrepresentation of paternal factors. Although recent studies highlight the influence of paternal health, lifestyle, and genetic factors on pregnancy outcomes, clinical practices remain largely centered around maternal health. This maternal focus overlooks the contributions of sperm quality, proteomic biomarkers, and paternal lifestyle factors in RPL, limiting the effectiveness of diagnostics and treatments [89,90].

Another limitation is the variability in diagnostic and assessment protocols across different institutions and regions, which complicates the ability to compare findings and develop standardized approaches. Proteomic analysis, for example, has shown promising results in identifying biomarkers for RPL, but it is still an emerging field with limited accessibility and high variability in methodology, making it challenging to implement universally [91].

The complexity of genetic and epigenetic interactions further complicates RPL research. While advanced genetic testing methods, such as next-generation sequencing, offer valuable insights, they also reveal a wide range of genetic variants, many of which lack established clinical relevance. This creates challenges in interpreting results, especially when counseling couples on the implications of detected variants. Additionally, the effects of environmental and lifestyle factors on epigenetic markers are not fully understood, particularly in how they might contribute to transgenerational risks [92].

### 7.2. Future Research Directions

#### 7.2.1. Expanded Focus on Paternal Contributions

Future research should place greater emphasis on paternal contributions to RPL, including comprehensive studies on sperm proteomics, genetic mutations, and lifestyle influences. By further exploring how specific sperm proteins and epigenetic alterations affect embryonic development, researchers can better understand the mechanisms through which paternal factors contribute to RPL. Additionally, developing targeted diagnostic protocols that consider both maternal and paternal health will help create more balanced and effective clinical assessments [93].

#### 7.2.2. Advances in Proteomic Biomarkers

Proteomics holds significant potential for identifying novel biomarkers in sperm that are linked to fertility and RPL risk. Future studies should aim to refine proteomic methodologies to improve consistency across studies and make proteomic analysis more accessible in clinical settings. Integrating proteomic data with other ‘omics’ technologies, such as genomics and epigenomics, could provide a more comprehensive profile of RPL risk factors, leading to highly personalized diagnostic and treatment options [94].

#### 7.2.3. Longitudinal and Multigenerational Studies on Environmental and Epigenetic Influences

Longitudinal studies are needed to assess how environmental exposures and lifestyle factors impact epigenetic markers in both parents over time. Furthermore, multigenerational studies exploring how these epigenetic changes are passed to offspring can provide insights into transgenerational risks associated with RPL. Understanding these intergenerational effects could lead to preventive strategies for individuals and families with a history of pregnancy loss [95,96].

#### 7.2.4. Standardization of Diagnostic and Intervention Protocols

There is a pressing need to establish standardized diagnostic protocols that can be universally applied in clinical practice to improve the comparability and reliability of findings across studies. Standardized protocols would help identify the most effective diagnostic markers and intervention strategies for RPL. Additionally, as genetic and proteomic technologies advance, developing guidelines for interpreting complex genetic and proteomic data will be critical for ensuring accurate counseling and treatment decisions [97].

#### 7.2.5. Microbiome Analysis in Paternal Contributions

The seminal microbiome has emerged as a critical factor influencing male infertility and potentially RPL. Studies indicate that an imbalance in seminal microbiota may be associated with reduced sperm quality, including DNA fragmentation and oxidative stress, both of which are key factors in RPL. For example, elevated levels of certain bacterial genera, such as *Flavobacterium* and *Lactobacillus*, have shown associations with abnormal sperm morphology and oxidative stress, respectively [98]. Moreover, research has highlighted that microbial communities within the uterine cavity and fallopian tubes may directly impact reproductive outcomes by altering the local immune response and epithelial interactions, crucial for successful sperm transport and embryo implantation [99]. For instance, the presence of certain microbes, including *Lactobacillus* and *Curvibacter*, has been linked to adverse outcomes in unexplained recurrent spontaneous abortion, suggesting that specific microbial compositions in these regions may disrupt normal fertility processes [100]. Future research should focus on characterizing these microbial profiles and investigating whether these bacteria directly influence sperm quality or cause reproductive dysfunction through inflammation [101]. Integrating microbiome data with genetic and proteomic profiles could also provide a more comprehensive understanding of RPL risk factors, potentially enabling more targeted and effective interventions.

#### 7.2.6. Integration of Psychosocial Support in Clinical Practice

The emotional toll of RPL is profound, yet psychological support is often secondary to clinical care. Future research should explore the benefits of integrating mental health support within RPL treatment programs. Studying the effects of counseling, stress management, and peer support groups on pregnancy outcomes and emotional well-being could offer more comprehensive care for couples facing RPL [102].

## 8. Conclusions

Paternal factors are increasingly recognized as pivotal contributors to recurrent pregnancy loss, with significant implications for reproductive health and pregnancy outcomes. This review underscores the complex mechanisms by which paternal age, genetic and epigenetic alterations, health conditions, lifestyle habits, and environmental exposures influence sperm integrity and embryonic development. The identification of novel sperm proteomic biomarkers offers promising avenues for improving diagnostics and tailoring personalized interventions in male infertility and RPL.

Addressing paternal risk factors through lifestyle modifications—such as smoking cessation, alcohol reduction, balanced nutrition, and appropriate physical activity—as well as medical treatments and environmental interventions is essential for reducing the incidence of RPL. Integrated approaches involving collaborative assessments of both partners, preventive strategies, and comprehensive genetic counseling can enhance clinical evaluations and provide better support for couples experiencing pregnancy loss.

Despite advances in understanding paternal contributions to RPL, limitations persist in current research and clinical practices, notably the underrepresentation of paternal factors in diagnostics and the lack of standardized protocols. Future research should broaden the focus on paternal influences, standardize diagnostic and intervention methodologies, and explore the integration of proteomic and genetic biomarkers into clinical practice. By adopting a comprehensive approach that considers both maternal and paternal factors, healthcare providers can more effectively support couples in achieving healthy pregnancies and improving overall reproductive outcomes.

## Figures and Tables

**Table 1:** Summary of novel sperm biomarkers: functions and clinical implications in recurrent pregnancy loss.

Biomarker	Function	Clinical Implications	References
ATP Citrate Lyase	Enzyme involved in lipid synthesis and energy metabolism	Elevated levels associated with RPL; may indicate metabolic disruptions affecting sperm quality and embryo development	[76]
DEAD-box Helicase 1	RNA helicase involved in RNA processing and gene expression	Overexpression may disrupt fertilization processes, leading to compromised embryo development and increased risk of RPL	[76]
Fatty Acid Synthase	Enzyme critical for fatty acid synthesis and membrane formation	Elevated levels linked to RPL; may affect sperm membrane integrity, impacting motility and fertilization capability	[76]
Histone 1.2	Nuclear protein involved in chromatin structure and regulation	Increased levels associated with RPL; suggests issues with chromatin remodeling that may compromise embryo stability	[76]
Hexokinase 1 (HK1)	Enzyme catalyzing the first step of glycolysis for energy production	Reduced levels observed in men with RPL; may indicate decreased sperm energy metabolism, affecting motility and viability	[77]
RuvB-like Helicase	Enzyme involved in DNA repair and maintenance of genomic stability	Deficiency linked to RPL; suggests a role in preserving sperm DNA integrity, critical for successful fertilization and embryo development	[77]

## Data Availability

No new data were created or analyzed in this study. Data sharing is not applicable to this article.
